# Supplementary figures and images for: Positive Correlation between Relative Concentration of Spermine to Spermidine in Whole Blood and Skeletal Muscle Mass Index: A Possible Indicator of Sarcopenia and Prognosis of Hemodialysis Patients
Source: Biomedicines. 2023 Mar 1;11(3):746. doi: 10.3390/biomedicines11030746 (PMC10045508; doi:10.3390/biomedicines11030746)

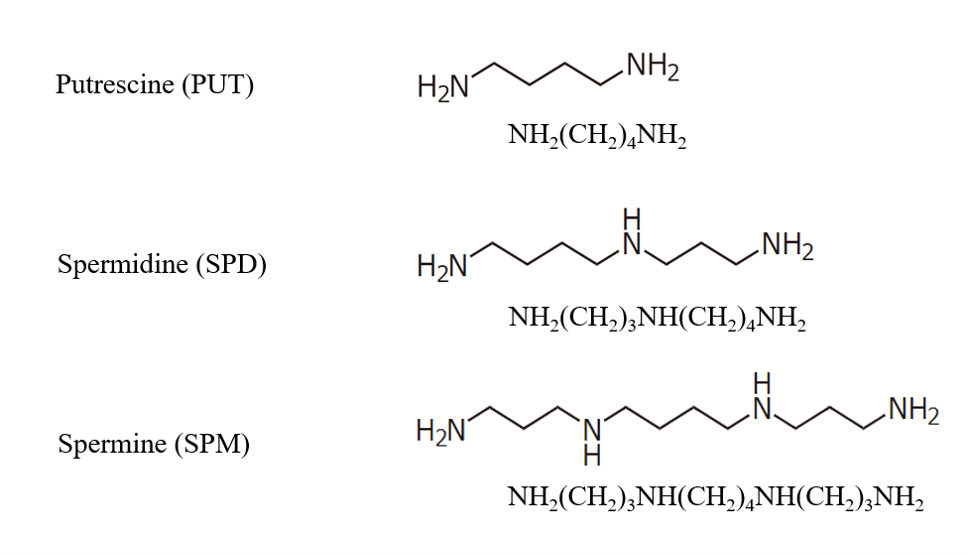

Supplement: Supplementary file 1 [file biomedicines-11-00746-s001.zip › Figure S1.tif]

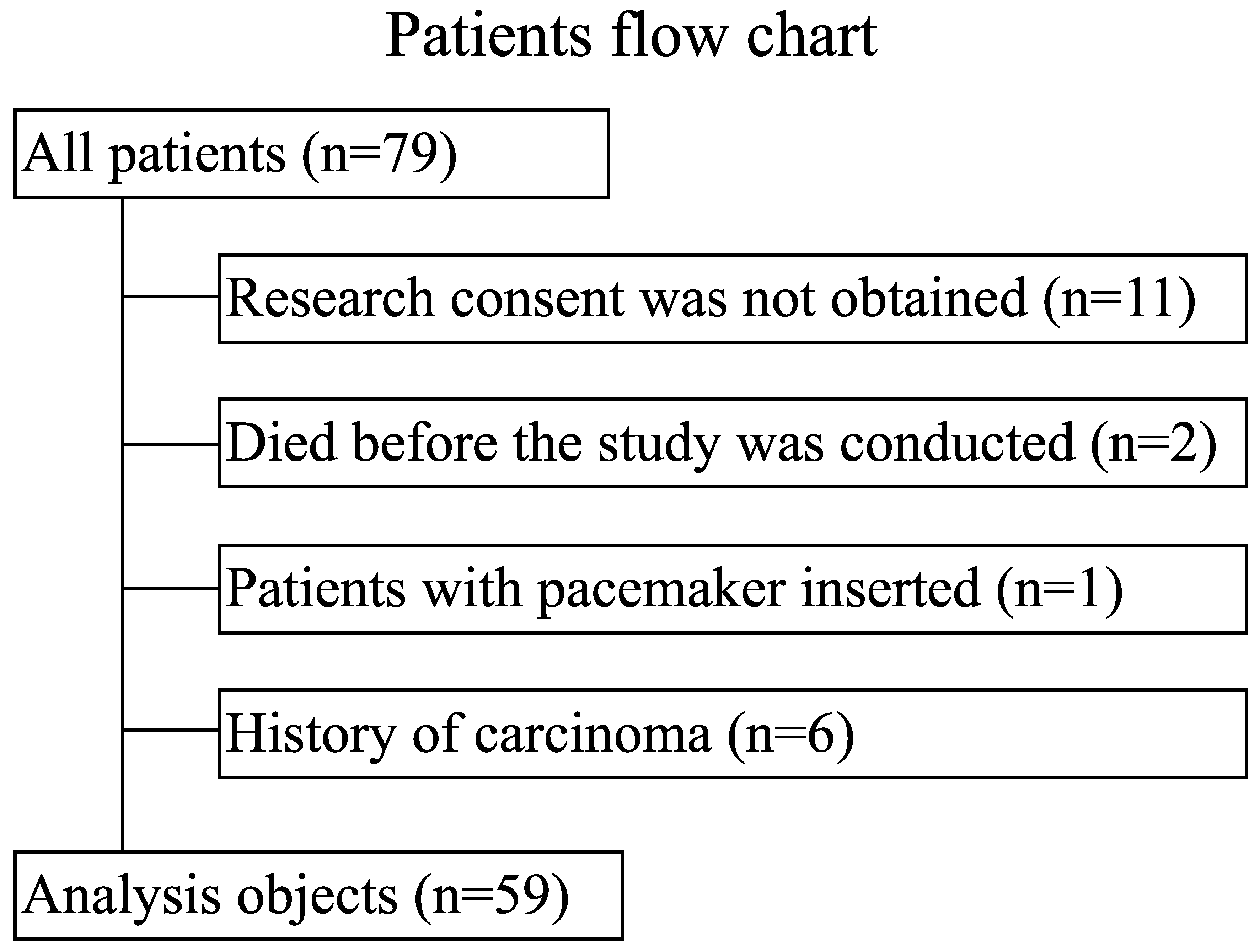

Supplement: Supplementary file 1 [file biomedicines-11-00746-s001.zip › Figure S2.tif]

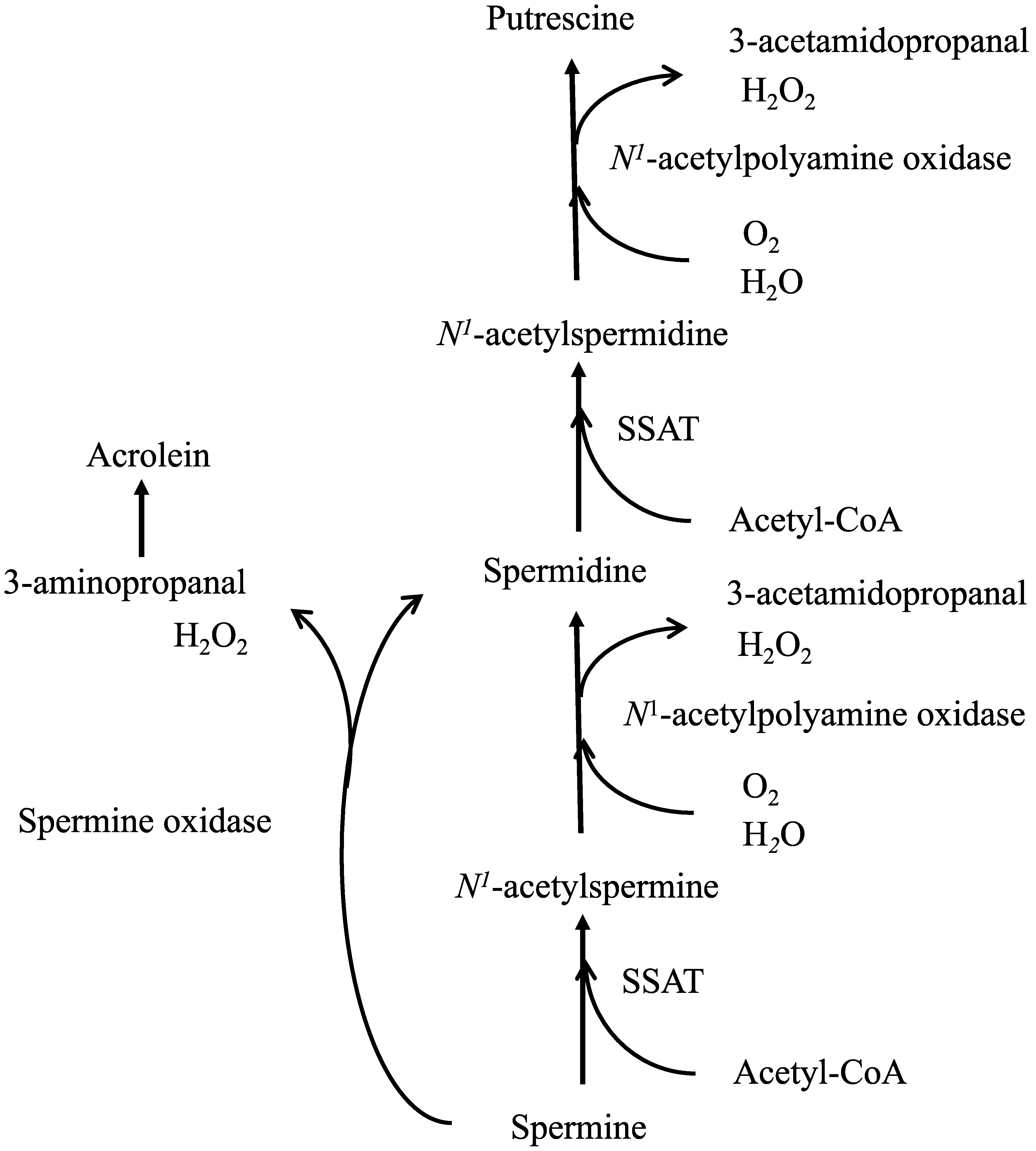

Supplement: Supplementary file 1 [file biomedicines-11-00746-s001.zip › Figure S3.tif]
